# Vitamin D and chronic kidney disease: Insights on lipid metabolism of tubular epithelial cell and macrophages in tubulointerstitial fibrosis

**DOI:** 10.3389/fphys.2023.1145233

**Published:** 2023-03-29

**Authors:** Luís Eduardo D. Gonçalves, Magaiver Andrade-Silva, Paulo José Basso, Niels O. S. Câmara

**Affiliations:** ^1^ Laboratory of Transplantation Immunobiology, Department of Immunology, Institute of Biomedical Sciences, University of São Paulo, São Paulo, Brazil; ^2^ Laboratory of Experimental e Clinical Immunology, Department of Clinical Medicine, Faculty of Medicine, Federal University of São Paulo, São Paulo, Brazil

**Keywords:** immune cell, renal fibrosis, beta-oxidation, VDR (vitamin D receptor), metabolism

## Abstract

Chronic kidney disease (CKD) has been recognized as a significant global health problem due to being an important contributor to morbidity and mortality. Inflammation is the critical event that leads to CKD development orchestrated by a complex interaction between renal parenchyma and immune cells. Particularly, the crosstalk between tubular epithelial cells (TECs) and macrophages is an example of the critical cell communication in the kidney that drives kidney fibrosis, a pathological feature in CKD. Metabolism dysregulation of TECs and macrophages can be a bridge that connects inflammation and fibrogenesis. Currently, some evidence has reported how cellular lipid disturbances can affect kidney disease and cause tubulointerstitial fibrosis highlighting the importance of investigating potential molecules that can restore metabolic parameters. Vitamin D (VitD) is a hormone naturally produced by mammalian cells in a coordinated manner by the skin, liver, and kidneys. VitD deficiency or insufficiency is prevalent in patients with CKD, and serum levels of VitD are inversely correlated with the degree of kidney inflammation and renal function. Proximal TECs and macrophages produce the active form of VitD, and both express the VitD receptor (VDR) that evidence the importance of this nutrient in regulating their functions. However, whether VitD signaling drives physiological and metabolism improvement of TECs and macrophages during kidney injury is an open issue to be debated. In this review, we brought to light VitD as an important metabolic modulator of lipid metabolism in TECs and macrophages. New scientific approaches targeting VitD e VDR signaling at the cellular metabolic level can provide a better comprehension of its role in renal physiology and CKD progression.

## 1 Introduction

Chronic kidney disease (CKD) is a long-term and gradual loss of kidney function. Inflammation and cellular metabolic imbalance are the main processes involved in kidney fibrosis, an inevitable pathological hallmark of all CKDs, characterized by the excessive accumulation of extracellular matrix components in the kidney. Tubulointerstitial fibrosis (TIF) refers to fibrosis outside of the glomerulus involving tubules and interstitium as a result of a sustained inflammatory response triggered by a particular kidney injury. Tubular epithelial cells (TECs) are considered the epicenter of renal damage during TIF development (Qi and Yang, 2018; Fu et al., 2022). TECs are important producers of pro-fibrotic and inflammatory mediators and can interact with immune cells in the interstitium potentiating the inflammatory response (Eardley et al., 2008; Feng et al., 2018; Conway et al., 2020).

Macrophages are immunological agents, and their presence in kidneys is considered a mixed blessing: while they are required for immune surveillance, they also play a pivotal role in both CKD occurrence and progression. Data from human and experimental observations show that macrophages are related to the severity of fibrosis (Yu et al., 2010), and it is possibly aggravated due to the direct communication with TECs (Komada et al., 2018; Jiang et al., 2022; Yang et al., 2023). TECs and macrophages undergo metabolic reconfiguration influencing inflammation and fibrosis development (Kang et al., 2015b; Bhatia et al., 2019). Lipid metabolism, specifically fatty acid oxidation (FAO, also referred to as β-oxidation), is the main pathway disturbed in TIF affecting primary TECs since they use the FAO as their primary energy source. In this line, macrophages rely on metabolic reprogramming to achieve an effector response (Van den Bossche et al., 2017; Dowling et al., 2021; Wculek et al., 2022) and the lipid metabolism status can determine the macrophage polarization toward a pro- or anti-inflammatory phenotype (Batista-Gonzalez et al., 2019). Cell metabolism has been addressed in recent studies to understand how nutrients can regulate function of certain cell subsets and, thus, change the course of diseases (Stine et al., 2022). The comprehension of how cell metabolism can orchestrate renal fibrosis offers potential strategies to disrupt CKD development and progression.

Vitamin D (VitD) is the “sunshine” vitamin due to it being synthesized in the skin under sunlight exposure. Its biosynthesis involves coordinating processes among the skin, liver, and kidneys (Bikle, 2014; Bikle and Christakos, 2020; Bouillon et al., 2022). CKD patients have a compromised VitD production that affects not only the kidney physiology, but also the systemic metabolism. Moreover, there is a diversity of studies that associate VitD deficiency and the development of pathological inflammation and renal fibrosis (LaClair et al., 2005; Del Valle et al., 2007; Cuppari and Garcia-Lopes, 2009; Nigwekar et al., 2012; Wang et al., 2012; Zhang et al., 2022; Dhillon-Jhattu et al., 2023). Factors associated with the internalization of precursors for active VitD synthesis, such as endocytic receptors, are reduced in the injured kidneys, which could explain its low levels in CKD patients (Toi et al., 2019; Wen et al., 2022). Nutritional aspects and low sunlight exposure can also aggravate this clinical scenario, increasing the risk of mortality in patients with end-stage renal disease, the most aggressive phase of CKD (Yoon et al., 2019).

Altogether, we aimed to gather recent data regarding VitD function in TECs and macrophages, highlighting their metabolic features to provide a more solid basis for future research in the field. We believe that unraveling the mechanistic roles of VitD and its receptor in the kidney can lead to the development of effective therapeutical strategies to treat CKD.

## 2 Vitamin D metabolism

**FIGURE 1:**
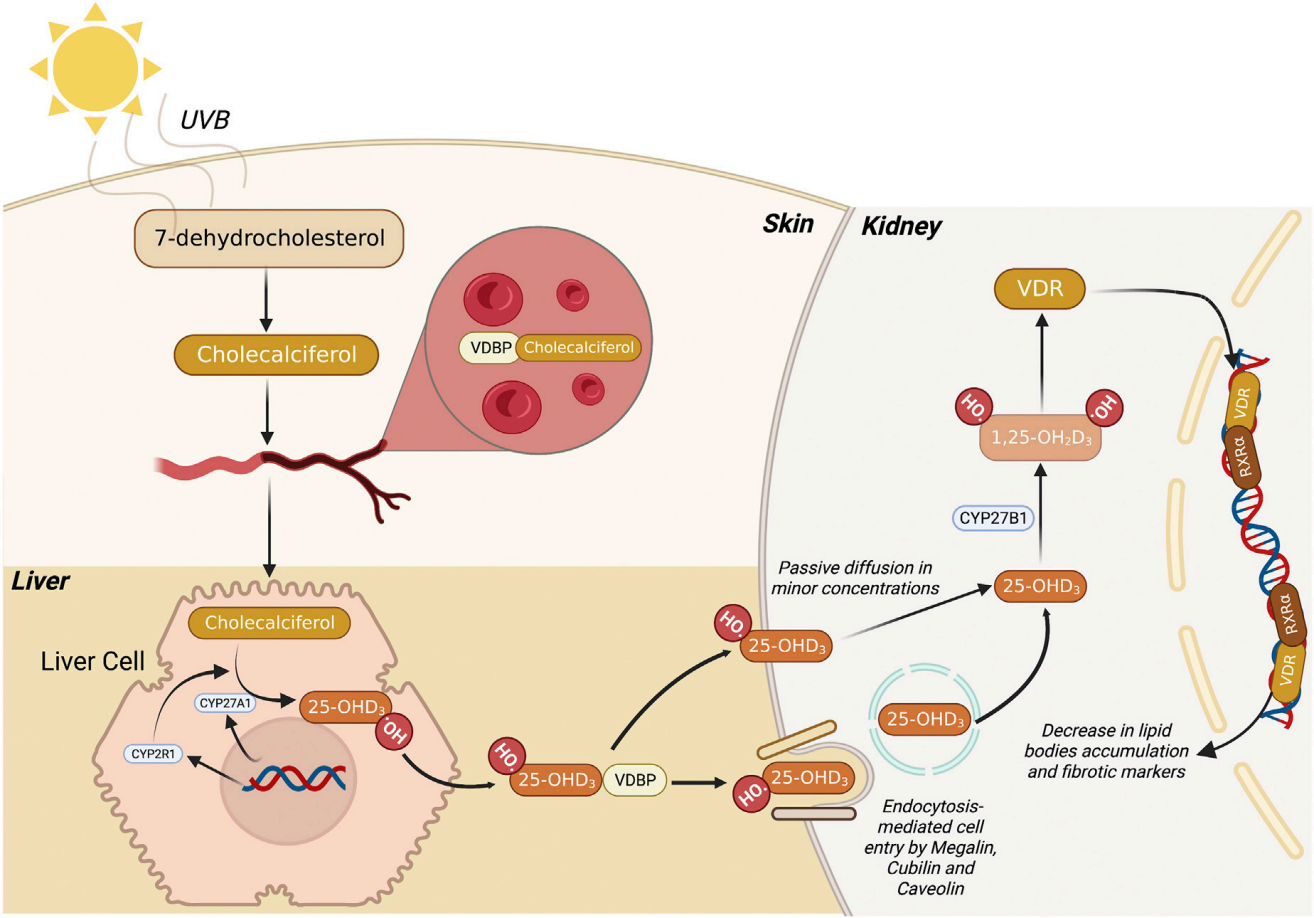
Metabolic route of 1,25 dihydroxyvitamin D3 synthesis. Firstly, 7-dehydrocholesterol (7-DHC) is converted to pre-vitamin D3 (Cholecalciferol) through the action of UVB light in the skin. Subsequently, this compound migrates to the bloodstream where it binds to VDBP, and the complex is transported to the liver. Then, the liver cell promotes the conversion of pre-vitamin D3 into 25-hydroxyvitamin D3 (25-OHD3) mediated by CYP27A1 and CYP2R1. Finally, 25-OHD3 migrates to the kidney where it can be passively diffused to the cells or be endocytosed *via* megalin, cubilin and caveolin membrane receptors. Once 25-OHD3 reaches the cytosol, it is converted by CYP27B1 into 1,25 dihydroxyvitamin D3 (1,25D3), the bioactive form of VitD. Subsequently, the 1,25D3 binds to VDR, migrate to the nucleus, and heterodimerizes with other nuclear hormones receptors, leading to the transcription of genes related to lipid metabolism. 7-DHC, 7-dehydrocholesterol; UVB, ultraviolet B (radiation); VDBP, vitamin D binding protein; VitD, vitamin D. Figure created with BioRender.com.

VitD is a sterol hormone that can be naturally produced in a multi-step, coordinated series of reactions among the skin, liver, and kidneys (Bikle, 2014; Bikle and Christakos, 2020; Bouillon et al., 2022). 7-dehydrocholesterol (7-DHC) is the precursor present in the skin compartment that, when irradiated by ultraviolet B (UVB) light (290–315 nm), is further converted into pre-vitamin D3, which temperature adjustments lead to the isomerization of pre-vitamin D3 into vitamin D3 (cholecalciferol) (Figure 1). Subsequently, vitamin D3 is transported to the liver through VitD Binding Protein (VDBP), where the next bioactivation takes place. In the liver, cytochrome P450 family members, such as CYP27A1 and CYP2R1, promote a hydroxylation reaction in carbon 25 of vitamin D3, yielding 25-hydroxyvitamin D3 (Calcitriol). The third and final step to produce the bioactive form of vitamin D is coordinated by kidney cells in which the 1ɑ-hydroxylase (CYP27B1) adds another hydroxyl group to carbon 1 of 25-hydroxyvitamin D3, leading to the synthesis of 1,25-dihydroxy vitamin D3 (1,25D3) (Wacker and Holick, 2013). The latter is responsible for the biological functions exerted through its biding to cognate VitD receptor (VDR) in the cytosolic compartment of a given cell. After being activated, the 1,25D3/VDR complex works as a transcription factor that operates along with another cytosolic receptor named Retinoid X Receptor α (RXR α ) (Barsony and Prufer, 2002).

Since 1,25D3 belongs to the fat-soluble and cholesterol-derived secosteroid family, it can diffuse through the cell bilayer membrane in minor concentrations. However, the primary process of 1,25D3 entering the cell is regulated by receptor-mediated endocytosis with the support of membrane proteins like caveolin, megalin, and cubilin (Trimarchi et al., 2021) (Figure 1). When present in the cytosol in its active form, 1,25D3 binds to VDR and translocate to the nucleus, where both VDR heterodimers and RXR α bind to the promoter region of VitD Responsive Elements (VDREs) on the DNA. The absence of VDR increases inflammation and aggravates inflammatory, metabolic, and autoimmune diseases, such as obesity, diabetes, vitiligo, inflammatory bowel diseases, and sepsis (Froicu et al., 2003; Wong et al., 2009; Mackawy and Badawi, 2014; Doss et al., 2015; Rao et al., 2019). Moreover, the beneficial role of VitD and VDR in renal inflammation and fibrosis have been reported in different studies described in the following topics.

### 2.1 Vitamin D in the kidney

In kidneys, VDR is expressed in the macula dense of the juxtaglomerular apparatus, glomerular parietal cells, podocytes, and proximal tubular epithelial cells (PTECs), evidencing a role for VitD in kidney homeostasis (Wang et al., 2012).

Patients diagnosed with CKD have a progressive reduction of VitD levels (LaClair et al., 2005; Nigwekar et al., 2012) and VitD deficiency is prevalent in 76.1% of stage 5 CKD patients submitted to the hemodialysis (Del Valle et al., 2007; Cuppari and Garcia-Lopes, 2009; Zhang et al., 2022; Dhillon-Jhattu et al., 2023). Studies demonstrated in experimental models and in CKD patients a lower megalin expression in the kidneys (Toi et al., 2019; Wen et al., 2022) and the absence of megalin in mice causes the inability of PTECs to capture the 25(OH)D3-VDBP complex, which in turn, are excreted in the urine, leading to a drastic reduction of plasma level of 25(OH)D3 and 1,25D3 (Nykjaer et al., 1999; Negri, 2006). Thus, megalin is a critical molecule in the indirect production of active VitD synthesis in the kidney, and its low expression in PTECs could explain the low levels of VitD in CKD patients.

The VitD deficiency contributes to fibrosis and increases intrarenal inflammation (Goncalves et al., 2014; de Braganca et al., 2018; Zhang et al., 2021b). These two pathological aspects have been linked to changes in lipid cell metabolism in renal fibrogenesis. Thus, VitD deficiency may cause a metabolic disturbance that leads to “fibroinflammation” in the kidneys.

#### 2.1.1 General aspects of lipid metabolism of tubular cell

Metabolism is the combination of biochemical processes at the cellular level to provide energy and substrates to the body and maintain tissue homeostasis. The metabolic state can influence a diversity of chronic inflammatory diseases. In CKD, changes in TEC’s lipid metabolism emerged as underlying mechanisms contributing to inflammation and renal fibrogenesis (Kang et al., 2015b; Han et al., 2016; Han et al., 2017). The molecular mechanisms that lead to metabolic disturbances in kidney cells and the investigation of putative molecules in restoring kidney function have become a spotlight of research in nephrology.

The energy source of kidney cells is specific. Podocytes, mesangial, and endothelial cells rely on the glycolysis (Forbes, 2016), while TECs, use FAO preferentially as the main metabolic pathway to obtain energy. FAO is considered one arm of oxidative phosphorylation (OXPHOS) since it fuels the tricarboxylic acid (TCA) cycle, which in turns, provides reducing equivalents to electron transport chain (ETC) to produce ATP. TECs represent about 90% of kidney mass (Chen et al., 2019) and require a high ATP demand to maintain their physiological function due to their intense and constant activity of transporting/reabsorption of solutes in the kidney (Kang et al., 2015b; Bhargava and Schnellmann, 2017; Han et al., 2017).

The disrupted FAO in the kidney (Kang et al., 2015b; Han et al., 2016; Han et al., 2017) causes lipotoxicity, inflammation, and epithelial-mesenchymal transition (EMT) (Kang et al., 2015b). EMT is a process in that TECs lose their epithelial phenotype and acquire mesenchymal characteristics associated with renal fibrosis during CKD. Transforming growth factor-β (TGF-β) is considered the critical regulator of EMT and fibrosis. TGF-β-induced tubular injury drastically reduces the key rate-limiting enzymes of FAO, carnitine palmitoyltransferases (CPT)-1a and -2a expression in TECs, and impairs adequate ATP production (Kang et al., 2015b). In contrast, CPT1a overexpression in TECs reduced the number of inflammatory macrophages and increased anti-inflammatory macrophages in the kidneys (see Section 3) (Miguel et al., 2021). VitD has the potential to reduce the production of inflammatory mediators, such as iNOS, COX2, IL-6, TNF, and MCP-1 through the NF-κB and impairs EMT of TECs submitted to noxious stimuli, such as 4-hydroxy-2-hexenal or LPS (Kim et al., 2013; Du et al., 2019). Considering that inflammation is a process highly associated with changes in cell metabolism, it brings to light the potential of VitD to regulate “metabolic inflammation” during CKD development (Marcotorchino et al., 2014; Kang et al., 2015a; Chang and Kim, 2016; Santos et al., 2017; Santos et al., 2018; Blajszczak and Nonn, 2019).

Beyond CPTs, other molecules indirectly influence FAO. sirtuin 1 (SIRT1), a member of nicotinamide adenine dinucleotide (NAD^+^)-dependent histone deacetylase, induces mitochondrial biogenesis by leading to deacetylation and activation of peroxisome proliferator-activated *γ* coactivator-1α (PGC-1α) (Rodgers et al., 2005; Gerhart-Hines et al., 2007), considered a key regulator of mitochondrial biogenesis, mitochondrial dynamics and mitophagy (Scarpulla, 2011). Moreover, PGC-1α and PGC-1α-peroxisome proliferator-activated receptor a (PPARα), in association, induces the transcriptional activity of FAO genes, including CPT1a. The overexpression of PGC-1α in TECs improves inflammatory and pro-fibrotic signaling in the mouse model fibrosis (Han et al., 2017) and reinforces the importance of restoring mitochondrial lipid metabolism in kidney fibrosis development.

#### 2.1.2 Vitamin D and renal fibrosis: Insights on lipid metabolism of tubular cells

Previous studies correlated VitD and metabolic diseases, such as obesity and type 2 diabetes mellitus (T2DM) (Candido and Bressan, 2014). In terms of obesity, where mitochondrial and lipid metabolism is mainly affected, experimental studies contribute to understand the mechanisms of VitD on the regulation of lipid disturbances. The VitD supplementation impairs the weight gain in the obesity mouse model induced by a high-fat diet (Marcotorchino et al., 2014). These effects are suggested to be linked with an increase of FAO through the upregulation of FAO-related genes, such as PGC-1α/β, PPARα and CPT1 isoforms (CPT1a and CPT1b) (Marcotorchino et al., 2014).

As discussed in the previous topic, recent findings suggested the effects of VitD on sirtuins (Thakran et al., 2013; Liu et al., 2020). The diversity of biological activities of sirtuins can be associated with their cellular distribution. The sirtuins can be found in the cytoplasm, mitochondria, and nucleus, and the same sirtuin can alter its cellular localization (Hong et al., 2020). While the nuclear SIRT1 plays roles in several transcriptional, posttranscriptional and posttranslational aspects of lipid metabolism, the SIRT3 controls many processes in mitochondria, such as antioxidant effects, autophagy, mitochondrial unfolded protein response, and several aspects of energy metabolism, including glycolysis, FAO, TCA cycle, OXPHOS, and ETC (Zhao et al., 2022). SIRT3 activation is beneficial in the context of kidney injury since its absence in unilateral ureter obstruction (UUO, classical animal model of renal fibrosis) caused exacerbated injury and fibrosis (Locatelli et al., 2020). The beneficial mechanism of SIRT3 is related to its controlling of mitochondrial enzyme acetylation (Zhang et al., 2021a). Acetylated proteins lose their catalytic function impairing normal mitochondrial metabolism. This is the case of some mitochondrial enzymes, such as pyruvate dehydrogenase E1α (PDHE1α), ATP synthase subunit O (ATP5O), and CPT1a disrupt OXPHOS because they decrease the ATP production, increase lipid bodies accumulation, and also increase glycolysis in kidney tubular cells during fibrotic process after acetylation (Zhang et al., 2021a). Recently, the induction of SIRT3 expression *via* VitD actions can reduce the inflammatory profile by impairing NLRP3 inflammasome activation in skin lesions (Dong et al., 2021). NLRP3 is a classical inflammasome reported to be activated by lipids in the cytoplasm and it has been associated with the renal fibrosis pathway (Jiang et al., 2012; Zanoni et al., 2017; Sokolova et al., 2020). Based on these studies, SIRT3 is reduced in renal fibrosis and may impair the CPT1a activity due to becoming acetylated (Zhang et al., 2021a). VitD may induce SIRT3 expression in TECs that could restore CPT1a activity, improving FAO and reducing lipid accumulation. The last event could prevent NLRP3 inflammasome activation.

Regarding the role of SIRT1 in the pathogenesis of CKD, its activation has a protective role in the disease progression by inactivating different profibrotic pathways, such as SMAD signaling. It contributes to mitochondrial biogenesis, which protects from fibrosis (Simic et al., 2013; Chuang et al., 2014; Huang et al., 2014; Liang et al., 2014; Wang et al., 2019; Hong et al., 2020). Studies in hepatocytes have also shown that VitD deficiencies lead to a reduction of SIRT1 expression (Yuan et al., 2022). Mechanistically, a chromatin immunoprecipitation assay demonstrated that the VDR binds to the promoter region of the SIRT1 gene, leading to its transcription. The absence of SIRT1 is also correlated with dysregulated glucose metabolism in hepatocytes as observed in glucose overproduction, glucose intolerance, and hepatic insulin resistance (Yuan et al., 2022). Furthermore, it was demonstrated that SIRT1 is required in autophagy and lipophagy, controlling the lipid droplet catabolism and FAO (Sathyanarayan et al., 2017). In streptozotocin-induced diabetic nephropathy (DN—a leading cause of CKD), the impairment of autophagy has been associated with the worst renal injury scenario, evidencing that autophagy is renoprotective in DN (Ding and Choi, 2015). Paricalcitol or VDR overexpression in mouse PTECs ameliorated the albumin excretion and tubular damage and reduced inflammatory parameters in DN mice by improving the autophagic process (Li et al., 2022). In one study, *in vitro* exposure of PTECs to high glucose conditions showed reduced both, Ca^+2^ concentration and activation of PRKAA1/AMPK. However, paricalcitol treatment was able to increase Ca^+2^ concentration and AMPK activation through CAMKK2/CaMKKβ, restoring autophagy (Li et al., 2022). It suggests that AMPK is a SIRT1 activator by providing NAD^+^, the major cofactor in the activity of the sirtuin (Canto et al., 2009).

**FIGURE 2:**
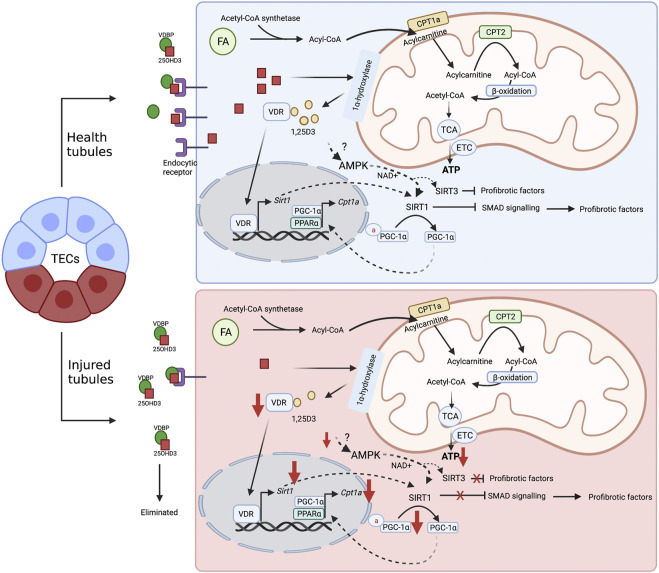
Proposal mechanism of VitD on healthy and injured tubular cell metabolism. FAO (or β-oxidation) is the main energy source of TECs. In the cytoplasm, FAs are transformed into Acyl-CoA by the enzyme Acyl-CoA synthetase. Then, the mitochondrial enzyme CPT1a converts acyl-CoA in acylcarnitine, which can be transported into mitochondrial matrix *via* carnitine/acylcarnitine translocase. Subsequently, acylcarnitine is subjected to the CPT2 activity to be reconverted into acyl-CoA and the FAO is initiated: acyl-CoA is converted into acetyl-CoA, which in turn, fuels the TCA cycle that provides reducing equivalents to ETC to produce ATP. VitD may be an important regulator of the FAO in TECs. After internalization *via* passive diffusion or receptor-mediated endocytosis, 25-hydroxyvitamin D3 is converted into its bioactive form, 1,25D3. The recognition of the active form by VDR causes transcriptional changes and leads to SIRT1 expression, which in turn, promotes the inhibition of profibrotic pathways, such as SMAD, and contributes to the deacetylation of PGC1 α and, thus, sustaining the FAO. In addition, VitD can also activate SIRT1 *via* AMPK activation, which increases NAD^+^ production. In the injured tubules the levels of endocytic receptors are reduced, impairing the optimal internalization of 25-OHD3 (calcitriol), and compromising the VitD activity. The reduced levels of VitD decrease the SIRT1 and SIRT3 expression as well as diminish the function of both PCG1 α and CPT1a. These alterations disrupt the FAO and contribute to profibrotic responses. 1,25D3, 1,25 dihydroxyvitamin D3; 25-OHD3, 25-hydroxyvitamin D3; AMPK, adenosine monophosphate-activated protein kinase; CPT, carnitine palmitoyltransferase; FA, fatty acids; FAO, fatty acid oxidation; PGC-1α, peroxisome proliferator-activated receptor-gamma coactivator-1α; TCA, tricarboxylic acid cycle; TECs, tubular epithelial cells; VDR, vitamin D receptor; VDBP, vitamin D binding protein. ETC, electron transport chain. Figure created with BioRender.com.

Altogether, it is plausible to suggest that SIRT1 and SIRT3 may play an important role in VitD effects in TECs, and the relationship among VitD-AMPK-sirtuins is an interesting issue for further investigations in tubular damage (Figure 2).

## 3 Vitamin D and macrophages in the kidney

Human and experimental CKD data have shown that TIF directly affects macrophage infiltration into the kidneys and interacts with renal cells (Eardley et al., 2008; Feng et al., 2018; Conway et al., 2020). TECs and macrophages interact directly and indirectly to potentiate inflammation in the kidneys. TECs are important C-C motif chemokine ligand 2 (CCL2) producers, a macrophage chemoattractant (Lv et al., 2018; Jia et al., 2022). Moreover, TEC-macrophages communicate by extracellular vesicles resulting in a negative feedback loop to promote renal inflammation and apoptosis in the mouse model of the fibrosis (Jiang et al., 2022). VitD-deficient animals increase the infiltration of inflammatory macrophages in the kidney, which reinforces that VitD is a good target to regulate inflammation and a unique molecule that can modulate different types of cell response. This topic will describe general aspects of macrophages and how metabolism can influence their effector response. Further, we describe the relationship between VitD/VDR and lipid metabolism, pointing out how VitD can be important in modulating metabolism and inflammation during renal fibrogenesis.

### 3.1 General aspects of macrophages: Polarization and metabolism

Macrophages are ubiquitous and sentinel cells that promote pathogen clearance and tissue homeostasis by recovering integrity during and after an inflammatory process (Wculek et al., 2022). Under physiological state, tissue-resident macrophages exert clearance functions and tissue maintenance, while in inflammatory conditions, bone marrow-derived monocytes migrate to the tissue, differentiate and polarize into M1 and after pathogen clearance reprograms into M2 macrophages to repair the inflammation-driven damage. Despite being classically subdivided into these two categories, it represents an oversimplification, due to the heterogeneity and complexity of macrophages in disease progression presented by different studies in the last years [revised by (Tang et al., 2019)].

The classically termed M1 macrophages, henceforward termed pro-inflammatory macrophages, are characterized by upregulation of antigen presentation markers, such as MHC-II and CD86. Furthermore, these cells have a vast repertoire of Pattern Recognition Receptors (PRRs) that activate several intracellular signaling pathways. Toll-like receptor 4 (TLR-4), a member of PRRs, for instance, drives the pro-inflammatory profile of macrophages because it leads to the activation of MyD88, which in turn, enables the activation of inflammatory factors like NF-κB, AP-1, and STATs (Kawai and Akira, 2010). As result, these M1 macrophages are able to produce pro-inflammatory mediators, such as IL-1, IL-6, IL-12 and TNF-α, increase the ROS production through NADPH oxidase, and enhance the iNOS expression (Panday et al., 2015; Van den Bossche et al., 2017).

Recently, it has been observed that macrophages rely on metabolic reprogramming to achieve an effector response (Van den Bossche et al., 2017; Dowling et al., 2021; Wculek et al., 2022). Pro-inflammatory macrophages profoundly change mitochondrial metabolism since the pyruvate is mostly redirected to lactate production (aerobic glycolysis or Warburg effect). Under the stimuli of LPS, rounded and circular mitochondria are observed in M1 macrophages, resulting from a process named mitochondrial fission (Kapetanovic et al., 2020). This rounded shape changes mitochondrial cristae structure and spatial organization that disrupts ETC, causing reduction in oxygen consumption (and consequently OXPHOS) and increased ROS formation (Yu et al., 2006; Buck et al., 2016).

In contrast with pro-inflammatory macrophages, M2 macrophages, henceforth named pro-resolving or anti-inflammatory macrophages, promote suppression of inflammation and tissue repair (Zhang et al., 2017). Pro-resolving macrophages are activated by IL-4 or IL-13 and participate in inflammation resolution by releasing anti-inflammatory cytokines, such as IL-10 and transforming growth factor β (TGF-β) (Viola et al., 2019). Moreover, they prioritize OXPHOS to drive their resolving functions. Upon IL-4 stimuli, for instance, macrophages upregulate arginase-1 expression that converts arginine into ornithine. Subsequently, ornithine enters the polyamines pathway through the action of ornithine decarboxylase, which leads to a putrescine formation and contribution of remodeling function by collagen matrix production and cell proliferation (Puleston et al., 2019). Simultaneously, changes in mitochondria also occur due to the upregulation of PPARγ, PGC1- α , and PGC-1β, which culminates in lipid accumulation and mitochondria biogenesis. Consequently, this mitochondrial lipid accumulation become a priority source for ATP production (Nomura et al., 2016). Importantly, PGC-1β activity also restrains the inflammatory profile of macrophages by inhibiting IL-6 and IL-12p40 production (Vats et al., 2006).

### 3.2 VitD and lipid metabolism of macrophages: Insights on kidney fibrosis development

**FIGURE 3:**
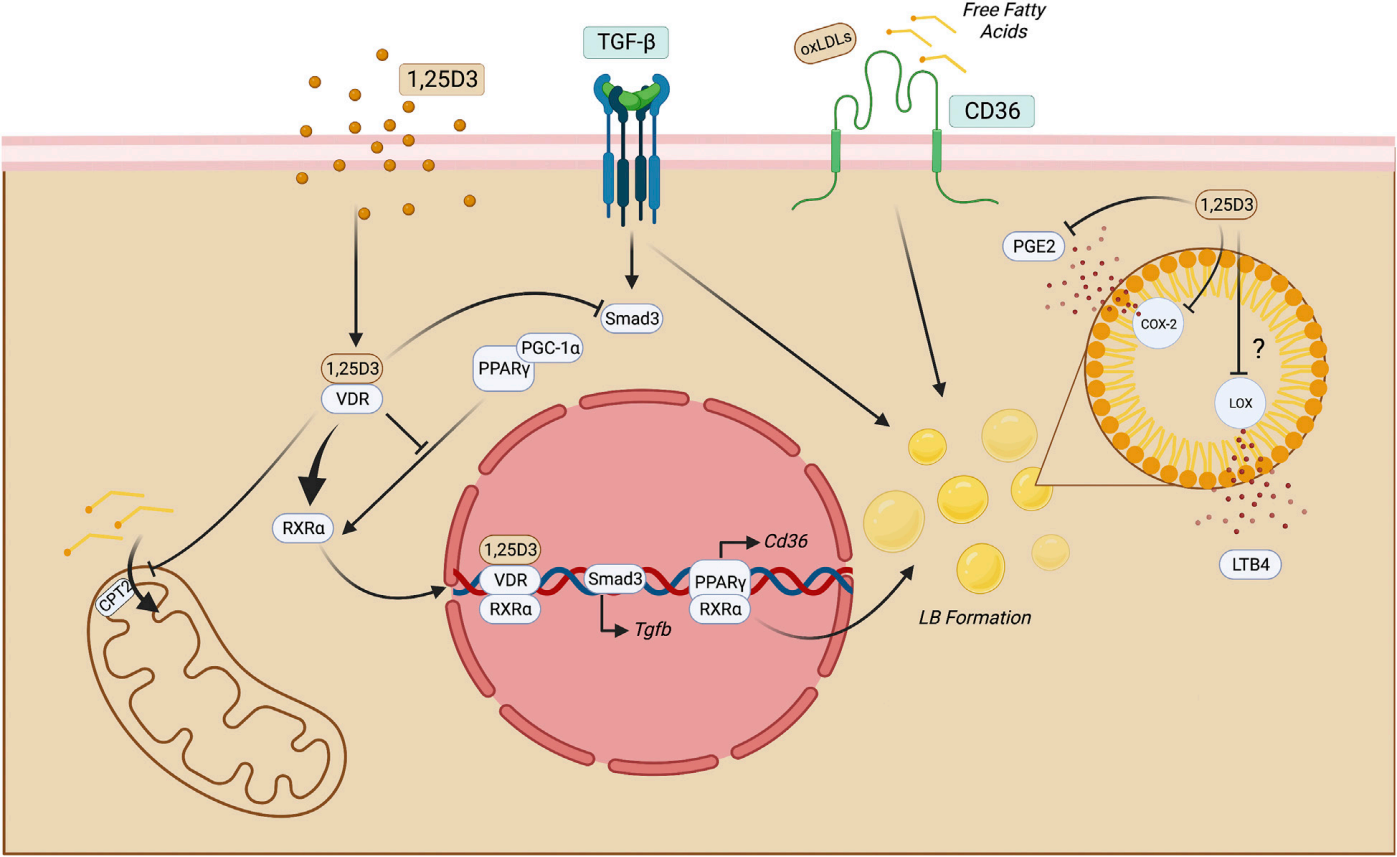
Putative mechanisms of 1,25D3 function in macrophages. Once 1,25D3 binds to VDR, it heterodimerizes with RXRα and translocate to the nucleus, impairing PPARγ-mediated functions. This may impact LB formation and, therefore, reduce the production of eicosanoids, such as LTB4 and PGE2. The latter may also be reduced through direct inhibition of COX-2 through 1,25D3. PPARγ blockade also may reduce CD36 exposure on the cell surface, leading to decreased lipid uptake. In macrophages, this process may limit FAO by inhibiting CPT2 and consequent long-chain fatty acid transport into the mitochondria. Moreover, since the fibrotic milieu is enriched with TGF-β, its signaling triggers Smad-3 and lipid accumulation in macrophages suggesting that this cytokine may have roles beyond fibrosis induction. 1,25 dihydroxyvitamin D3; COX-2, cicloxygenase-2; LOX, lipoxygenase; CPT, carnitine palmitoyltransferase; FAO, fatty acid oxidation; LB, lipid bodies; LTB4, leukotriene B4; PGE2, Prostaglandin E2; TECs, tubular epithelial cells; VDR, vitamin D receptor. Figure created with BioRender.com.

1,25D3 has been suggested as an essential regulator of lipid metabolism in macrophages in atherosclerosis and *Mycobacterium tuberculosis* infection. In both conditions, macrophages display a foamy aspect which indicates lipid accumulation, forming intracellular organelles termed lipid bodies (LBs). LB formation can be promoted by PPARγ translocation to the nuclear compartment and the function of these *bona fide* organelles is context dependent. In *M. tuberculosis*, the infection may be a source of neutral lipids to the bacteria, but also can regulate macrophage immune function (Knight et al., 2018). In atherosclerosis, oxidized LDLs (oxLDLs) are suitable activators of PPARγ and are uptaken by the scavenger receptor CD36 that induces profibrotic genes that contribute to thrombus formation (Nagy et al., 1998; Feng et al., 2000). 1,25D3/VDR complex leads to VDR-RXR complex activation, inhibits PPARγ-mediated function in the cell, and decreases LB synthesis in these macrophages. 1,25D3 may also limit the biogenesis of eicosanoids like prostaglandin E2 (PGE2) and leukotriene B4 (LTB4) in LBs since they produce these mediators. This directly impacts the inflammatory response because the PGE2 synthesis can modulate the antimicrobial activity of human macrophages by downregulating cathelicidin, reducing their capacity to eliminate intracellular pathogens. The mechanism that coordinates this function is E Prostanoid 2-mediated (EP2), a PGE2 receptor (Wan et al., 2018). In this context, it may suggest that 1,25D3 decreases PGE2 production, while enhancing antimicrobial peptide production. In contexts where CKD may take place due to obesity or hyperlipidemia, free fatty acids are uptaken by CD36 in the kidney by macrophages and this may cause lipotoxicity and triggering of inflammatory processes (Gai et al., 2019). Regarding LTB4 synthesis, it is still an open gap in macrophage lipid metabolism in CKD.1,25D3 may be suggested as a compound that can attenuate lipid accumulation and therefore prevent lipotoxicity since it can reduce LB formation and CD36-mediated lipid uptake (Figure 3).

VDR also controls M2 phenotype as studies showed that the deletion of this nuclear receptor in F4/80^+^ macrophages could decrease Arginase-1 and RELM-α expression and produce IL-13 after local injury (Zhang et al., 2014). In CKD, macrophages display a pro-resolving profile that relies mainly on OXPHOS, and enzymes like COX-2 are activated at high levels in infiltrating macrophages in the kidney tissue. 1,25D3 can reduce COX-2 expression in macrophages by reducing its expression and limiting eicosanoids’ availability to be converted (Wang et al., 2014). Furthermore, 1,25D3 can also counteract lipid accumulation through the downregulation of CD36 and upregulation of PPARγ and CPT1, which mediates the transport of long-chain fatty acids into the mitochondria and leads to FAO (Marino et al., 2022). Therefore, 1,25D3 may alleviate fibrosis by preventing lipid accumulation in anti-inflammatory macrophages that are pivotal cells in the fibrosis maintenance (Zhu et al., 2021). A fibrotic kidney may also benefit from 1,25D3 action due to its activity on PPARγ, which has a PGC-1ɑ as a coactivator. It has been shown that the coordinated action of these transcription factors can induce mitochondrial FAO enzymes in 3T3-L1 cell lines (Vega et al., 2000). Since 1,25D3 inhibits PPARγ translocation to the nucleus, it may also hamper the co-activation of PGC-1ɑ in pro-resolving macrophages in the kidney. However, it requires further evaluations.

Furthermore, endogenous PGE2 can enhance IL-33 production after LPS stimulation through the EP2 receptor (Samuchiwal et al., 2017). This cytokine belongs to the IL-1 family and is present in the context of tissue injury. Moreover, it may suggest that 1,25D3 binding to VDR could downregulate IL-33 synthesis by preventing lipid accumulation and PGE2-mediated action.

Another cytokine produced by anti-inflammatory macrophages is TGF-β which promotes fibroblast proliferation and proline production. This amino acid is essential for collagen biosynthesis (Liu and Chen, 2022). TGF-β is critical to the chronic stage of kidney diseases and activates Smad transcription factor family (Tang et al., 2021). One study showed that VDR could inhibit TGF-β -Smad signaling through direct contact with Smad3, which alleviated the fibrosis in the kidneys (Ito et al., 2013). It has been shown that macrophage-secreted TGF-β also induces LB accumulation in the cytosol of macrophages (D’Avila et al., 2011). Therefore, it can be suggested a dual protection during CKD mediated by 1,25D3: 1) reduction of the macrophagic fibrotic capability and 2) reductions of lipid accumulation by various mechanisms, including TGF-β-induced LB formation (Figure 3). However, it remains an open experiment to be tested in the context of CKD.

During CKD such as systemic erythematosus lupus nephritis, a bioinformatic study showed that several metabolic pathways are upregulated, including OXPHOS, sphingolipid, and glycerol metabolism (Cheuk et al., 2021). Similar pathways were observed in ischemia-reperfusion injury (IRI), in which biosynthesis of unsaturated fatty acids and glycerol metabolism were upregulated. The authors observed that TGF-β signaling was upregulated, which is expected in fibrotic kidney conditions. This may correlate with lipid metabolism-enriched pathways (Cheuk et al., 2021). Increased lipid metabolism in CKD can be observed, which participates in disease progression. 1,25D3 may be contributing to alleviating CKD through inhibition of lipid metabolism in these cells by blocking PPARγ translocation to the nucleus, CD36 exposure on the cell surface, and TGF-β-mediated LB synthesis. Experiments can be provided to test this in future studies by integrating the impact of 1,25D3 on the lipid interface in CKD macrophages.

## 4 Final remarks and future perspectives

Disturbances in the metabolic pathways cause fibroinflammation and progressive loss of kidney function. The development of safe and effective therapeutic strategies depends on a better understanding of how these effects are initiated and maintained. The reduction of VitD levels and its receptor expression may be determinant on the outcome of CKD and other inflammatory diseases. Thus, further studies regarding the beneficial role of VitD and VDR will support this hypothesis. In addition, the bidirectional relationship between renal and immune cells, especially TECs and macrophages, may rely on their metabolism, which the modulation by using VitD and other metabolism-based therapeutic strategies can provide clinician’s better management of CDK patients.
